# Diagnosis of lung squamous cell carcinoma based on metagenomic Next-Generation Sequencing

**DOI:** 10.1186/s12890-022-01894-3

**Published:** 2022-03-27

**Authors:** Ping Wei, Yang Gao, Jing Zhang, Jianlong Lin, Huibin Liu, Keqiang Chen, Weikai Lin, Xiaojia Wang, Chune Wang, Chao Liu

**Affiliations:** 1 Fujian University of Traditional Chinese Medicine, Fuzhou, 350122 Fujian China; 2 The Second Affiliated Hospital of Fujian Traditional Chinese Medical University, Fuzhou, 350003 Fujian China; 3 School of Biological Science and Medical Engineering, Beihang University, Beijing, China; 4 Hangzhou Matridx Biotechnology Co., Ltd, Bd 2-4, 2073 Jinchang Rd, Hangzhou, 311100 Zhejiang China; 5 Director of Respiratory Department, The Second Affiliated Hospital of Fujian Traditional Chinese Medical University, Fuzhou, 350108 Fujian China; 6 Director of Medical Department, Hangzhou Matridx Biotechnology Co., Ltd, Hangzhou, 311100 China

**Keywords:** Metagenomic Next-Generation Sequencing, Lung abscess, Lung squamous cell carcinoma, Pulmonary infection, Copy number variation

## Abstract

**Background:**

The clinical treatment of patients suspected of pulmonary infections often rely on empirical antibiotics. However, preliminary diagnoses were based on clinical manifestations and conventional microbiological tests, which could later be proved wrong. In this case, we presented a patient whose initial diagnosis was lung abscess, but antibiotic treatments had no effect, and metagenomic Next-Generation Sequencing (mNGS) indicated presence of neoplasm.

**Case presentation:**

A 62-year-old female was diagnosed with lung abscess at three different health facilities. However, mNGS of bronchoalveolar lavage fluid did not support pulmonary infections. Rather, the copy number variation analysis using host DNA sequences suggested neoplasm. Using H&E staining and immunohistochemistry of lung biopsy, the patient was eventually diagnosed with lung squamous cell carcinoma.

**Conclusions:**

mNGS not only detects pathogens and helps diagnose infectious diseases, but also has potential in detecting neoplasm via host chromosomal copy number analysis. This might be beneficial for febrile patients with unknown or complex etiology, especially when infectious diseases were initially suspected but empirical antibiotic regimen failed.

## Background

The radiological signs of lung abscess and lung cancer share several similarities, making accurate diagnosis difficult. In particular, lung abscess is an infectious disease that is characterized by the presence of pus-filled cavity [1]. On the other hand, up to 22% of primary lung cancers display similar, but relatively less cavities [2]. When cavitary lesions are observed in chest CT, clinicians usually consider the possibility of lung abscess since it is more common [3]. Initial diagnoses regarding pulmonary diseases are often empirical, which depend on conventional microbiological tests, CT scan and serological biomarkers for inflammation and tumor. However, these tests may lead to obscure, or even false diagnosis. For example, fastidious microorganisms are difficult to culture and thus are more challenging to identify. Moreover, when certain symptoms are shared by multiple diseases, the preliminary diagnoses might be inaccurate and thus delays effective treatment. Therefore, a comprehensive and unbiased microbiological test such as mNGS might be beneficial in cases where empirical treatment fails.

Metagenomic sequencing is a culture-free and hypothesis-free diagnostic method that sequences both microbial and host DNA/RNA directly from samples. It has been shown to detect pathogens from a variety of sample types including tissue [4], bronchoalveolar lavage fluid (BALF) [5], and peripheral blood [6].

## Case presentation

### Clinical presentations

On July 20th, 2020, a 62-year-old female was admitted to the affiliated hospital of Fujian University of Traditional Chinese Medicine. The patient had experienced cough, expectoration, and hemoptysis for one week and fever for three weeks. The patient had hypertension for 6 months but no difficulties in breathing and reported no prior history of allergy or smoking. One month ago, she visited a local hospital and the blood test showed 19.83 × 10^9/L WBC, 85% GR, 108.3 mg/L CRP and 0.056 ng/mL PCT. She was diagnosed with lung abscess in the left lower lobe. Therefore, piperacillin and levofloxacin were administered. However, three weeks prior to admission, the patient started to experience fever with a highest armpit temperature of 40 °C. The patient was then transferred to another two hospitals, where clinicians maintained the diagnosis of lung abscess. Antibiotics were adjusted to moxifloxacin and cefotaxime but with no relief of symptoms.

### Physical examination

Upon admission, the patient’s body temperature was 37.5 °C, respiration rate was 18/min, blood pressure was 102/71 mmHg, heart rate was 89/min. The patient was conscious with normal nutritional status.

### Diagnostic assessment

The blood test showed WBC of 30.64 * 10^9/L, Neutrophil of 25.94 * 10^9/L (86.1%), CRP of 72.67 mg/L, and PCT of 2.25 ug/L. The serological tests including G test, GM test, TB antibody test, SARS-CoV-2 antibody test, HIV, HCV, HBsAG and TP tests were all negative. Biochemistry analysis and BNP level were normal, with coagulation D-D concentration of 0.74 mg/L. As for tumor markers, the CEA was 8.68 ng/mL, the SCC was 3.10 ng/mL, and the CYFRA21-1 was 6.37 ng/mL.

**Fig. 1:**
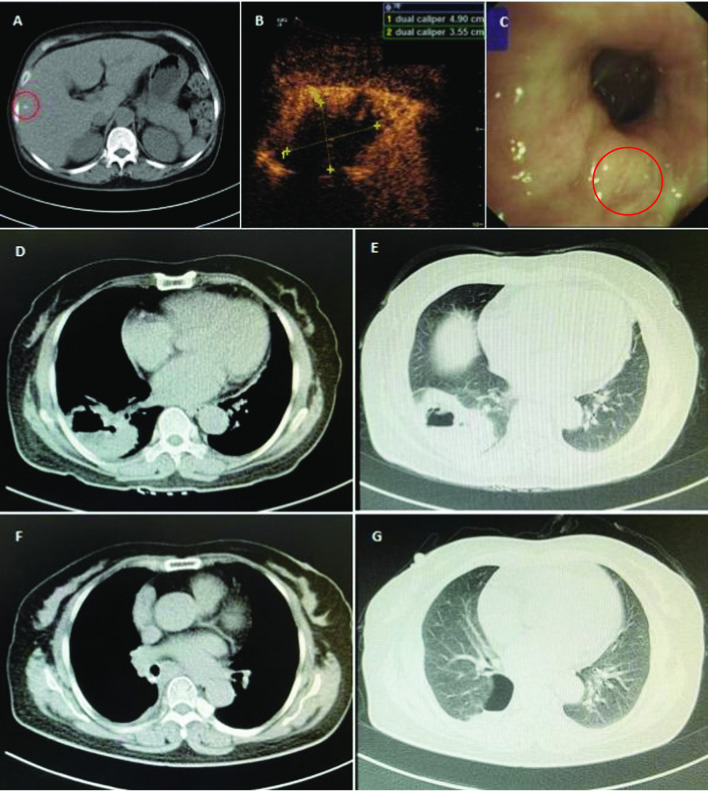
CT scan images. Abdominal CT images taken before surgery, and the circled area showed a small nodule (**A**). Chest color Doppler ultrasound images, the size of the pulmonary lesion was 4.9 cm × 3.55 cm, and necrotic tissue was shown in the center of the lesion (**B**). Bronchoscopy image of the lower right lobe of bronchial lumen, the circled area showed white mucus (**C**). Chest CT images taken before surgery (**D**, **E**). Chest CT images taken on follow up visit in September (**F**, **G**)

The patient’s abdominal CT scan revealed small nodules in the bile duct and hepatic cysts (Fig. 1A, red circle). Chest color Doppler ultrasound showed mixed-echo pattern in the right lower lobe (Fig. 1B). No neoplasia, foreign body or bleeding were observed in the lumen of the left and right main bronchus and segmental bronchus of each lobe. A small amount of white mucus was seen in the bronchial lumen of the dorsal segment of the lower right lobe (Fig. 1C, red circle). Chest CT scan showed highly concentrated masses (5.5 cm × 8.69 cm) on the inferior lobe of right lung and cavitary lesion, which was the main basis for the diagnosis of abscess (Fig. 1D, E). The tracheoscopy showed that the main trachea was unobstructed with no secretions. The mucus culture reported *Candida* species and BALF culture reported *Aspergillus* species. Blood and pus cultures were negative.

### Initial treatment

Vancomycin was given for three days, but the patient remained febrile with an elevation of WBC, GR and CRP levels (Table 1). The treatment was then adjusted to voriconazole and meropenem. WBC and GR decreased but still higher than normal (Table 1).

**Table 1:** Clinical record of the patient during treatment

Date	Infectious indicators	Treatment	Body temperature (highest)
7.10	WBC: 19.83 * 10^9/L ↑ GR: 85% ↑ CRP: 108.3 mg/L ↑ PCT: 0.056 ng/mL	Before hospitalization	40.0 °C
7.20	WBC: 30.13 * 10^9/L ↑ GR: 86.1% ↑ CRP: 72.67 mg/L ↑ PCT: 2.25ug/L ↑	Vancomycin	40.3 °C
7.21	CEA: 8.68 ng/mL ↑ SCC: 3.10 ng/mL ↑ CYFRA21_1: 6.37 ng/mL ↑		
7.23	WBC: 30.64 * 10^9/L ↑ GR: 90.3% ↑ CRP: 149.24 mg/L ↑	+ Voricanozole	
7.24	Metagenomic Next-Generation Sequencing result indicated tumour, clinicians performed lung puncture	Meropenem	40.0 °C
7.28	WBC: 19.37 * 10^9/L ↑ GR: 85.9% ↑ CRP: 153.02 mg/L ↑	+ Voriconazole	
	Report of lung puncture showed tumor, SLC was considered		
7.29	CEA: 9.59 ng/mL ↑ SCC: 4.3 ng/mL ↑		37.5 °C
7.30	Operation (radical resection of lung cancer)		
7.31	WBC: 9.62 * 10^9/L ↑ GR: 76.9% ↑ CRP: 124.36 mg/L ↑ PCT: 0.35%↑	Cefoperazone sodium sulbactam	
8.13	Discharged from hospital		
9.8	WBC: 7.19 * 10^9/L GR: 57.3% CRP: 20.1 mg/L ↑ PCT: 0.22% CEA: 1.84 ng/mL SCC: 0.4 ng/mL CYFRA21_1: 1.9 ng/mL		Normal (< 37 °C)

*WBC* white blood cell, *GR* granulocytes, *CRP* C-reactive protein, *PCT* plateletcrit, *CEA* carcinoembryonic antigen, *SCC* squamous cell carcinoma, *CYFRA21_1* cytokeratin-19 fragment

“↑” indicates higher than normal range

### mNGS testing

**Fig. 2:**
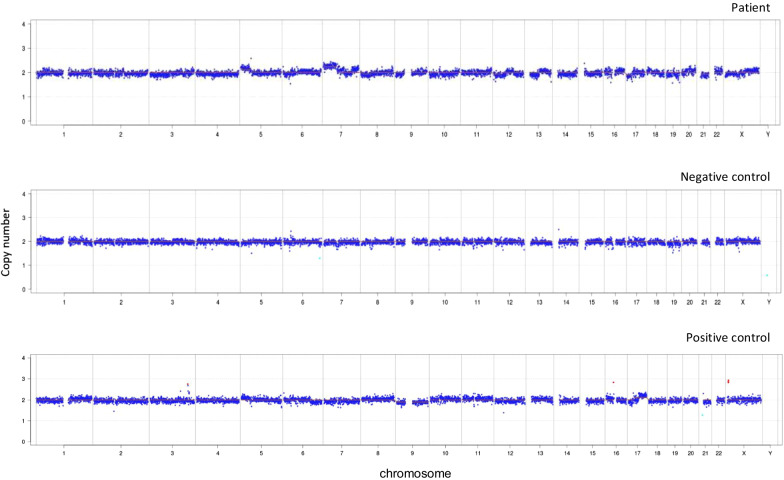
Results of chromosomal copy number analysis. The negative control was the peripheral blood obtained from a healthy individual with no known inherited diseases. The positive control was the BALF sample obtained from a patient diagnosed with lung adenocarcinoma

Due to the unsuccessful treatment, the patient’s BALF was sent for mNGS. No pathogens were detected other than common respiratory colonizers, indicating an etiology of non-infectious diseases (Table 2). Moreover, mNGS data also suggested the presence of malignant tumor, which was based on the copy number variation (CNV) analysis of the host DNA. Using a read depth-based approach [7], the normalized copy number of the patient showed abnormalities (duplications and deletions) on several chromosomes when compared to a healthy individual, which was likely caused by chromosomal instabilities of tumor cells (Fig. 2).

**Table 2:** Microorganisms detected by BALF mNGS

Genus				Species		
Type	Name	Sequence reads	Relative abundance%	Name	Sequence reads	Relative abundance%
G−	*Prevotella*	4144	47.12	*Prevotella denticola*	2124	24.15
G+	*Streptococcus*	1316	14.96	*Streptococcus sanguinis*	443	5.04
G−	*Veillonella*	985	11.20	*Veillonella parvula*	365	4.15
G+	*Rothia*	349	3.97	*Rothia mucilaginosa*	111	1.26
G+	*Actinomyces*	214	2.43	*Actinomyces oris*	50	0.57
G−	*Haemophilus*	187	2.13	*Haemophilus parahaemolyticus*	134	1.52
G+	*Lactobacillus*	173	1.97	*Lactobacillus salivarius*	124	1.41
Fungus	*Candida*	9	0.10	*Candida albicans*	9	0.10
Fungus	*Nakaseomyces*	2	0.02	*[Candida] glabrata*	2	0.02

### Follow-up and outcomes

**Fig. 3:**
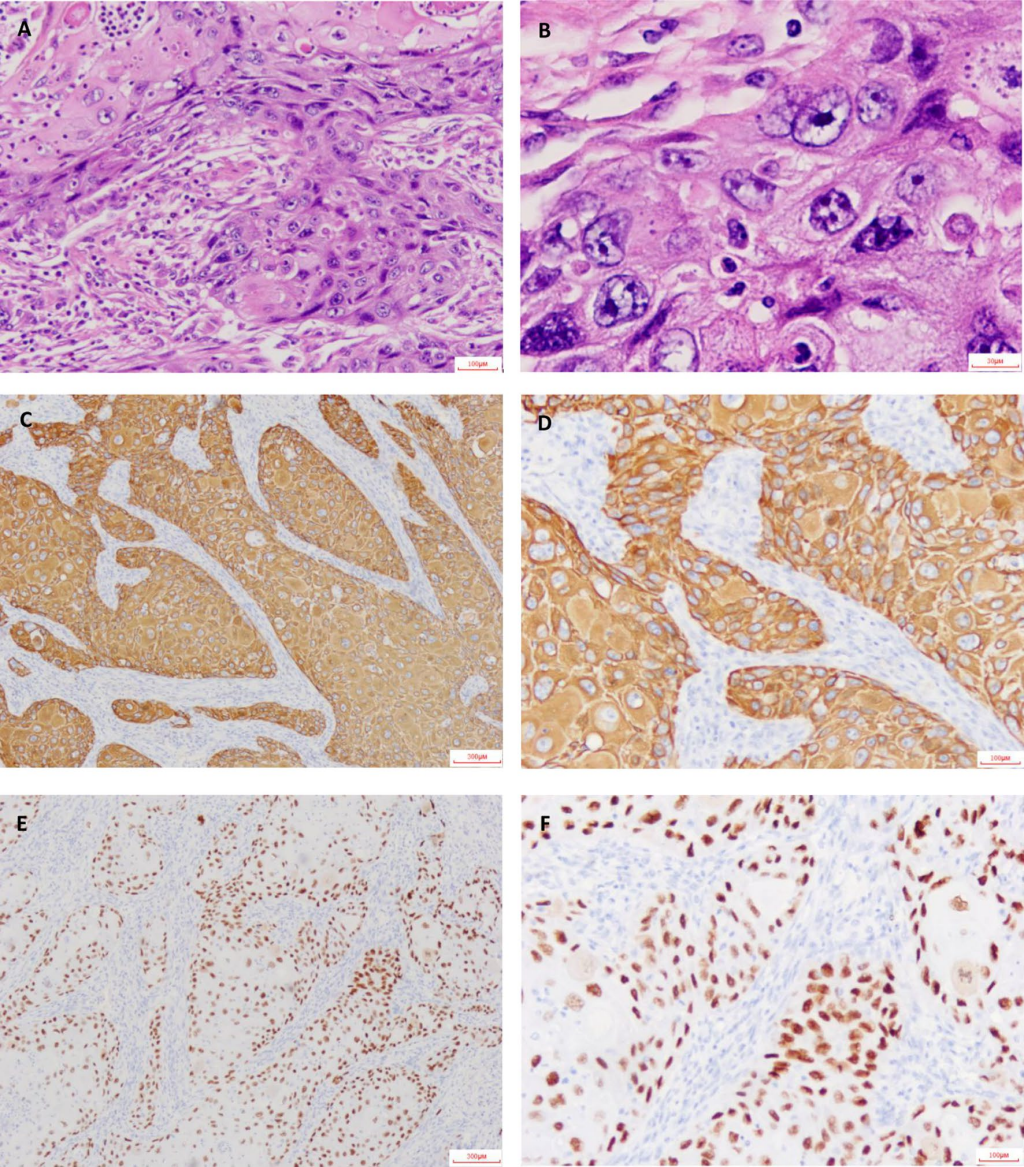
H&E staining and immunohistochemical staining. H&E stain was shown as 100× (**A**) and 400× (**B**). CK5/6 stain was shown as 40× (**C**) and 100× (**D**). P63 stain was shown as 40× (**E**) and 100× (**F**). Microscope type: OLYMPUS BX43; acquisition software: LOGENE-I Image Processing System

Clinicians performed percutaneous transthoracic lung biopsy of the abscess and collected 20 mL of fluid. Thinprep liquid-based cytology combined with H&E staining were carried out, and malignant tumor cells were seen (Fig. 3A, B). Immunohistochemistry further supported the diagnosis of lung squamous cell carcinoma (LUSC) (Fig. 3C–F). The patient received radical resection of tumor and was given cefoperazone sodium sulbactam. One month following the surgery, the serological level of tumor markers (CEA, SCC, CYFRA21-1) returned to normal, and pulmonary lesions were largely removed (Table 1, Fig. 1F, G).

## Discussion and conclusions

Prior to mNGS, microbial infection was suspected due to the results of conventional tests, including CT scan, culture, blood, and serology test. However, neither *Candida* nor *Aspergillus* spp. reported by culture were common causes of lung abscess. In addition, although the patient showed increased levels of tumor markers, lung cancer was not considered since abscess and inflammation were known to cause elevation of these markers.

The results of mNGS did not support microbial infections. The criteria for which microorganisms were called was described in one of our previous studies [8]. First, it did not detect *Aspergillus* while only one colony was seen in culture. Aspergillosis was then ruled out due to the negative G and GM test; second, both mNGS and culture detected *Candida albicans*. Nevertheless, *Candida* species are commonly seen in BALF [9], and in most cases cause no health problems; Third, empirical antibiotics failed to alleviate the symptoms.

Abnormal copy number on the chromosomal level is suggestive of hereditary disorders or tumor-induced genomic instability. Several techniques have been developed to analyze CNV, including PCR-based [10], hybridization-based (immunofluorescence, Giemsa, southern blotting, etc*.*) and microarray-based methods [11]. Among these, hybridization-based methods are complex to perform with low resolution. As a result, PCR or microarray-based approaches are commonly used. However, mNGS has the distinctive advantage over these methods since it can provide both microbiological and copy number information in a single test [12]. In our case, DNA sequences were aligned to the human hg19 (GRCh37) reference genome. Guanine-cytosine (GC) content bias was corrected using LOESS regression. Standard deviation of the read fold change of each bin of data (bin size 100 k) and normalized read counts were obtained. Copy number variation was called based on XHMM [13] and Canoes [14] (using reference data of normal chromosomal copy numbers). Due to the fact that tumor cells were mixed with normal cells in the BALF, the overall copy number change was sometimes difficult to observe, for which we employed a computerized algorithm to predict the possibility of neoplasm according to one of our previous studies [15]. The large CNVs (> 10 M) encompassing several chromosomes were unlikely to be caused by inherited disorders. Instead, these CNVs were indicative of tumor cells [16]. Although the detection of these chromosomal aberrations does not render a definitive cancer diagnosis, it prompted clinicians to conduct more focused diagnostic testing for tumors instead of infectious diseases. As a result, this case highlights a novel and important role for mNGS to assist in cancer diagnosis. If tumor is not of primary concern, clinicians may not proceed to perform biopsy or pathological analysis, which could lead to delay of effective treatment. It has been reported that up to 19% of fever of unknown origin (FUO) could be attributed to cancer [17].

In our case, lung abscess was considered at first according to clinical diagnosis. The level of the tumor markers was slightly elevated, but infection can also cause an increase of these markers. Moreover, the other examination and clinical manifestations did not show strong evidence of tumor. On the other hand, fever, elevated level of hemogram and inflammatory markers, and CT images were more consistent with the pathology of abscess. Therefore, although tumor was not entirely excluded, the initial diagnosis was abscess, and other tests were not performed for further verification.

Since the success of treatment and prognosis depends on how early the cancer is diagnosed, the patient would significantly benefit from a timely diagnosis.

On the other hand, mNGS testing also comes with limitations such as cost and turnaround time. It is only recommended for patients suspected of microbial infections who are in severe conditions or when initial treatment fails. The CNVs detected by mNGS can only suggest the presence of tumor DNA but could not clearly indicate the location or whether the tumor is benign or malignant and therefore should be corroborated with pathological tests.

## Data Availability

The fastq file generated by mNGS is available in National Center for Biotechnology information, Sequence Read Archive. (direct link: https://www.ncbi.nlm.nih.gov/bioproject/PRJNA743985; accession number: PRJNA743985). The reference genome human hg19 (GRCh37) can be accessed from the National Center for Biotechnology information, Assembly database. (direct link: https://www.ncbi.nlm.nih.gov/assembly/GCF_000001405.13/, GenBank assembly accession: GCA_000001405.1; RefSeq assembly accession: GCF_000001405.13).

## Abbreviations

mNGS: Metagenomic Next-Generation Sequencing
LUSC: Lung squamous cell carcinoma
CNV: Copy number variation
WBC: White blood cell
GR: Granulocytes
CRP: C-reactive protein
PCT: Procalcitonin
CEA: Carcinoembryonic antigen
SCC: Squamous cell carcinoma
CYFRA21_1: Cytokeratin fragment 19
